# The Effect of Movement on Cognitive Performance

**DOI:** 10.3389/fpubh.2018.00100

**Published:** 2018-04-20

**Authors:** Raed Mualem, Gerry Leisman, Yusra Zbedat, Sherif Ganem, Ola Mualem, Monjed Amaria, Aiman Kozle, Safa Khayat-Moughrabi, Alon Ornai

**Affiliations:** ^1^Ramat Zevulun High School, Ibtin, Israel; ^2^Oranim Academic College, Qiriat Tivon, Israel; ^3^The National Institute for Brain & Rehabilitation Sciences-Israel, Nazareth, Israel; ^4^Faculty of Health Sciences, University of Haifa, Haifa, Israel; ^5^Safed Academic College, Safed, Israel

**Keywords:** sequential memory, attention, cognition, movement, walking, Bloom’s taxonomy, mathematics achievement

## Abstract

The study examines the relationship between walking, cognitive, and academic skills. Students from elementary, middle, high school, and college were required to walk for 10 min prior to completing feature detection, Simon-type memory, and mathematical problem-solving tasks. Participants were counterbalanced to remove a time bias. Ten minutes of walking had a significant positive effect on Simon-type memory and critical feature-detection tasks among all age groups. Separately, with mathematical problem-solving ability, higher performing high-school students demonstrated significant positive effects on mathematical reasoning tasks based on the Bloom Taxonomy. However, poorly achieving high-school students performed significantly better than those with higher grades in mathematics on tests of mathematical problem-solving ability based on the Bloom’s Taxonomy. The study indicates that there is justification to employ relatively simple means to effect lifestyle, academic, and cognitive performance.

## Introduction

Brain plasticity and cognitive function are significantly improved by physical activity (1). The importance of this association is more important than ever given the significantly increased sedentary behavior and obesity worldwide in childhood and adulthood and the upsurge in aging populations of diminishing fitness and cognitive functions (2). Kramer and Erickson (3, 4) assessed the hypothesis that exercise and physical activity might protect and even augment brain and cognitive function throughout one’s lifespan. They critically reviewed the literature on the relationship between physical activity on brain and cognition function. The authors included prospective observational or epidemiological studies; non-human animal studies; and randomized human clinical trials. They reported that the literature supports the assertion that cognitive and brain function is enhanced by physical activity.

McDonnell et al. (5) found that a single 30-min period of physical activity is related to increases in neuroplasticity with discernible positive effects in motor-skill coordination and in declarative memory. Hillman et al. (6) studied the relationship between electrophysiological variables associated with attention and treadmill walking on academic performance. Hillman and colleagues observed the cognitive function and cardiopulmonary fitness at rest of each of their participants to determine baseline aerobic and cognitive capability. An exercise period was provided that involved 20 min of walking on a treadmill at 60% of the estimated maximum heart rate followed by the assessment of cognitive function once heart rate resumed to within 10% of pre-exercise levels. The participants exhibited a significant increase in response accuracy; greater P3 event related potential amplitude, and significantly improved performance on academic achievement tests after aerobic exercise when compared with rest. The results of Hillman and colleague’s study revealed that exercise is associated with increases in cognitive function as measured by tests of academic performance and attention. These data suggest that even single sessions of light exercise can influence essential processes related to cognitive skills.

Smith et al. (7) required adults in their late 1920s and early 1930s to ride exercise bicycles for a period of 30 min. Brain-state changes immediately after the session were examined and again after 15 min. Short-interval intra-cortical inhibition before and at 0 and 15 min following 30 min of ergometer cycling at low-moderate or moderate-high intensity was examined as well as cortical stimulus-response curves (90–150% resting motor threshold). Neuroplasticity, it was concluded, could be improved by a single 30-min session of physical activity with noticeable changes after only 15 min of exercising.

Neuroplasticity is associated with the both the actual number as well as the connection strength between neurons as well as in regional connectivities within the brain (8). It is on this basis that we can explain why it is that exercise and physical activity positively increase cognitive–motor function. Kamijo et al. (9) had studied the relationship between academic achievement and physical exercise, as did Hillman et al. (10). Of interest is the fact that Cotman and Berchtold (11) noted that with physical activity, brain-derived neurotrophic factor (BDNF), that supports synaptogenesis in the basal forebrain and hippocampus, functions in areas fundamental to memory, learning, and thinking and is significantly elevated in rodents. Similar findings have been found in humans (12). Interestingly, although BDNF increases in mouse hippocampus after 7 days of wheel running compared with sedentary mice (11), termination of the exercise according to Ref. (13) reverses, in rodents, the increased cell number, and cognitive gains. Chaddock-Heyman et al. (14) found advantage from regular exercise for the brain’s white matter and noted that connectivities between diverse cortical gray matter brain regions were facilitated. The relationship between brain state and an individual’s physical fitness was observed in 24 9- and 10-year-old children. Researchers observed thicker and denser white matter among those children exhibiting greater degrees of physical fitness in turn linked with significantly superior attention span, memory, and cognitive facilities.

In the present, we examined the relationship between 10 min of walking to determine its effects on cognitive and academic performance in various age groups.

## Materials and Methods

### Feature Detection and Simon-Type Memory

#### Participants

Seventy-five elementary school children all in grade 5 consisting of 35 females and 40 males and 75 middle school children all in grade 7 (36 females and 39 males), 75 high school all in grade 9 consisting of 38 females and 37 males and 58 college students (35 females and 23 males) between the ages of 22 and 27 years participated in the study. All participants came from middle class backgrounds as determined by the methodology of Hollingshead and Redlich (15) IRB approval was obtained from the Research Committee of Oranim Academic College.

In the mathematic problem-solving study 75 high-school students in grade 9 consisting of 38 females and 37 males participated in the study. IRB approval was obtained from the Research Committee of Oranim Academic College.

#### Procedure

Participants were divided into two groups. Both groups were given a sequential memory task consisting of a Simon-type multisensory memory game (16) and a feature-detection task consisting of presentations of famous paintings twice, once with the complete painting and later with a minor feature of the painting missing. The task of each participant was to find the missing feature. Group A was given the tasks immediately after 10 min of outdoor walking in the school yard at a normal pace at 10 a.m. each session and Group B was given the tasks without 10 min of walking. A period of 10 min of walking was chosen rather than 15 min as longer periods would likely covert aerobic into anaerobic metabolism (17). After a period of a full week, Group B performed 10 min of walking prior to the task and Group A did not. Therefore, the groups were counterbalanced. This design attempted to avoid time as a confounding variable. In all cases, both the Simon-type and feature-detection tasks, all participants in both pre- and post-walking sessions were counterbalanced in that in the pre-testing situation Group A was examined prior to walking, and B after walking, with different but statistically equivalent tasks.

In the mathematical problem-solving task, participants were divided into two groups. Both groups were given a mathematical problem-solving task based on PISA, a multinational test of mathematical problem-solving ability (18). The tasks were based on Bloom’s Taxonomy (remember, understanding, apply, and analyze) (18, 19).

We did not use the PISA test itself but rather employed “PISA-type” questions that were not part of current PISA tests but rather examples of questions typically found on PISA. We were not testing academic achievement but rather changes in mathematical problem-solving ability as measured by these question examplars—after aerobic walking.

As all groups received equivalent questions, PISA standardization became irrelevant in the context of the present study as the entire test was not used but only samples. The focus was not the PISA test results but rather changes in how the participants answered the same type of questions reflecting mathematical problem-solving ability.

Group A performed the PISA tasks immediately after 10 min of walking and Group B was given the tasks without 10 min of walking. After a period a full week, Group B had 10 min of walking prior to the task and Group A did not. Therefore, the groups were counterbalanced. This design attempted to avoid time confounded the results. This design attempted to avoid time as a confounding variable. All participants in both pre- and post-walking sessions were counterbalanced in that in the pre-testing situation Group A was examined prior to walking, and B after walking, with different but statistically equivalent tasks.

Each level of the taxonomic hierarchy contained two questions and was taken from the last edition of PISA. The questions provided the participants both before and after the walking intervention were different but statistically equivalent in difficulty. During the week between pre- and post-testing, participants had no opportunity to practice the material contained in PISA, as it was not part of their curriculum for their grades. Participants, therefore, had no practice effect. Questions were taken from the Israel Arabic edition of PISA from the ministry of education with questions exemplified in the materials provided by the OECD (20).

### Statistical Analysis

Paired *t*-test comparisons were performed between pre- and post-walking tasks as each participant served as his own control. Non-parametric Spearman’s rho correlations were also performed in order to examine the impact of attention on memory in the Simon-type and feature-detection tasks.

## Results

### Simon-Type Memory and Feature Detection

The analysis created forced dichotomies between poor and high-performing students in mathematical problem solving. Weak students had a grade average of less than 60% and those defined as having a grade point average of greater than 60%.

The results demonstrated significant improvement in the final PISA scores with walking of 10.6% (*t* = 5.01; df, 31; *p* < 0.0001) and sequential memory tasks with an 11% increase in performance (*t* = 5.727; df, 74; *p* < 0.0001) after 10 min of walking. Among middle school participants, significant improvement was recorded on feature detection with a 26% increase in performance (*t* = 2.39; df, 74; *p* < 0.05) and sequential memory tasks with a 15.4% increase in performance (*t* = 2.905; df, 74; *p* < 0.001) after 10 min of walking. Among high-school participants, significant improvement was recorded on feature detection with a 25% increase in performance (*t* = 3.48; df, 69; *p* < 0.001) and on sequential memory tasks with an 8% increase in performance (*t* = 3.48; df, 69; *p* < 0.001) after 10 min of walking. Among college-aged participants, significant improvement was recorded on feature detection with a 34% increase in performance (*t* = 5.05; df, 57; *p* < 0.001) and on sequential memory tasks with a 20% increase in performance (*t* = 11.98; df, 57; *p* < 0.001) after 10 min of walking. The tabular representation of the results may be found in Tables 1 and 2.

**Table 1:** Simon-type memory task.

Age		Before	After	% Change of the mean	*t*	*p*-Value	*N*
Elementary school	Mean	7.18	8.08	11.13	5.7278	0.0001	75
	SD	2.10	1.99				
Middle school	Mean	9.15	10.82	15.49	2.9059	0.0043	75
	SD	3.12	3.59				
High school	Mean	11.65	12.67	8.01	3.4877	0.0009	75
	SD	2.68	2.84				
College	Mean	9.98	12.54	20.43	11.9825	0.0001	58
	SD	2.55	2.95				

**Table 2:** Feature detection.

Age		Before	After	% Change of the mean	*t*	*p*-Value	*N*
Elementary school	Mean	30.50	45.00	32.22	5.7029	0.0001	75
	SD	20.78	30.23				
Middle school	Mean	55.63	75.63	26.45	2.3952	0.0195	75
	SD	31.77	36.16				
High school	Mean	51.21	68.10	24.81	3.4877	0.0004	70
	SD	27.66	34.31				
College	Mean	71.59	108.15	33.80	5.0585	0.0001	58
	SD	33.08	42.19				

Table 3 reports Spearman’s rho correlational data for memory and feature-detection tasks. For all age groups, no significant relationships were noted, indicating the independence of the experimental tasks and the collective effects of walking on performance.

**Table 3:** Spearman’s rho correlation coefficient.

NS	Correlation Coefficient	0.070	Elementary school
NS	Correlation coefficient	0.028	Middle school
NS	Correlation coefficient	0.154	High School
NS	Correlation coefficient	0.206	College

### Mathematical Skill

**Figure 1:**
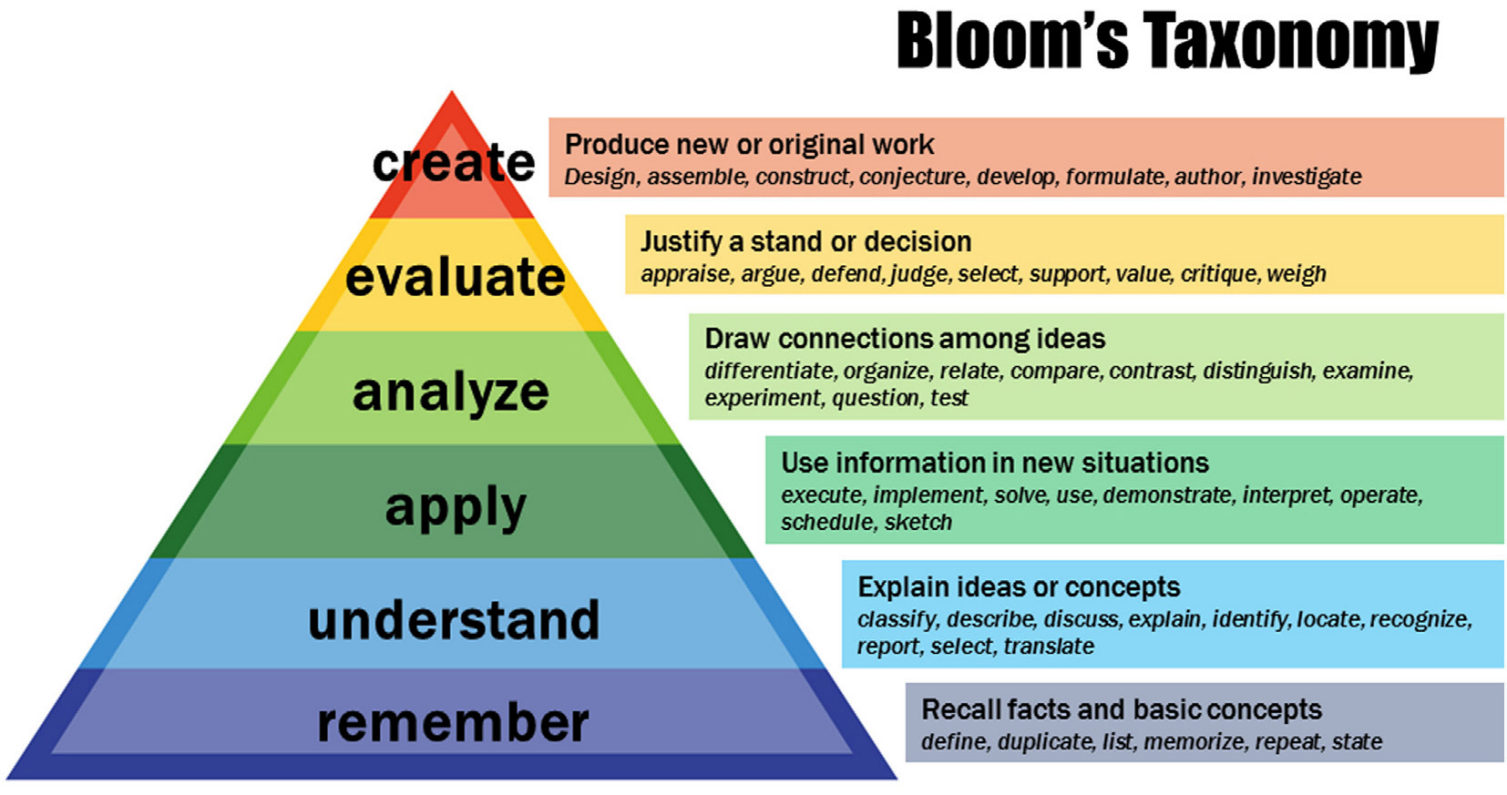
Representation of Bloom’s Taxonomy in the cognitive domain. The taxonomy provides a hierarchical model in order to categorize learning objectives in levels of specificity and complexity. Represented here is the knowledge-based cognitive domain that includes: *remember* (characterized by knowledge of specifics -terminology, specific facts, and ways); *understanding* (employing means of dealing with specifics them and knowledge of the universals and abstractions in a field such as understanding of facts and ideas by organizing, comparing, translating, interpreting, giving descriptions, and stating the main ideas); applying (involving acquired knowledge—e.g., problem solving in new situations); analyzing or breaking knowledge down into component parts; evaluating which is creating informed judgments and validating ideas and create which is the ability to create new knowledge.

The present study additionally aimed to determine the effects of 10 min of walking on the effectiveness of mathematical learning performance in high-school male and female students. Participant performance was based on the Bloom’s Taxonomy hierarchy that is represented in Figure 1.

#### Participants

Seventy high-school students all in grade 9 (M = 17 years), consisting of 38 females and 37 males, participated in the study. Each participant served as his/her own control. A mathematical test based on the Bloom Taxonomy was employed (19). Students were examined prior to and after a 10-min period of walking. The following week each participant, at the same time, repeated a comparable task after a 10-min walk, and performance was re-examined. This then eliminated a temporal bias in the results and the groups were counterbalanced. The high-school level participants were divided into two groups, one consisting of low achievers, defined as those participants with a grade average of less than 60%, and a second group consisting of high achievers, whose grade point average was greater than 60%.

#### Results

The academically lower group obtained a significant increase of 6.29% in *remember* and 11.1% in *understanding* on the PISA tasks that evaluated performance within the Bloom’s Taxonomy. *Applying* demonstrated an 8.4% increase and 19.6% in *analyze* domains. The final grades for all participants increased by 10.6%. The results for the low-achieving group are represented in Table 4.

**Table 4:** Students with low achievement in mathematics.

		Remember	Understanding	Apply	Analyze	Final grade
Before	Mean	12.50	10.29	9.29	7.64	39.71
	SD	1.70	1.64	1.68	1.15	5.46

After	Mean	13.29	11.43	10.07	9.14	43.93
	SD	1.98	1.87	1.77	1.61	6.07

% Change of the mean		6.29%	11.11%	8.46%	19.63%	10.61%

*t*		2.474	3.6632	3.2937	3.4963	5.0182

*p*-Value		0.0279	0.0029	0.0058	0.0039	0.0002

The academically higher achieving group obtained a significant increase of 4.4% in *remember* and 3% in *understanding* on PISA tasks that evaluated performance within the Bloom’s Taxonomy. *Applying* demonstrated an 8.0% increase and 6.9% in *analyze* domains. The final grades for all participants increased by 5.5%. The results for the low-achieving group are represented in Table 5.

**Table 5:** Students with high achievement in mathematics.

		Remember	Understanding	Apply	Analyze	Final grade
Before	Mean	19.00	17.92	17.15	15.54	69.62
	SD	2.24	2.29	2.79	3.69	9.65

After	Mean	19.85	18.46	18.54	16.62	73.46
	SD	2.27	2.11	3.13	3.15	9.27

% Change of the mean		4.45%	3.00%	8.07%	6.93%	5.52%

*t*		4.429	2.5011	4.4538	2.8094	5.3812

*p*-Value		0.0008	0.0279	0.0008	0.0158	0.0002

## Discussion

Movement in the form of aerobic walking significantly enhances performance of children on tests of sequential memory requiring cognitive–motor interaction and on tests of feature detection associated with attentional focus. Older children demonstrated significantly greater improvement in sequencing tasks than did younger children and these children, in turn, performed significantly better on tasks requiring attentional focus. The results should generate both future research and impact on the nature of classroom instruction in the context of the relationship between movement and cognition.

Postural muscles, we have claimed elsewhere (21), were the chief channels for the evolution of motor and cognitive binding. For a more comprehensive review of the nature of evolutionary brain development, posture, brain size, and the implications for limitations of the pelvis as well as the genetic implications, the reader is referred to Melillo and Leisman (22) and Falk et al. (23).

Reduced postural activity in childhood harms natural exploration of the surrounding environment, thereby reducing the ability to learn from experiences. Deviations from normal postural development or from normal levels of postural activity can disrupt or delay cerebellar and cortical maturation and may disrupt the underlying oscillatory timing mec (24); hanisms on which motor and cognitive binding is based (25–29). As a result, cognition, more likely, evolved secondarily and in parallel to the evolution of human upright bipedalism. Although viewed as separate functions historically, it can be argued that complex motricity and cognition are functionally connected. The effect of this is that motricity is inextricably involved in cognitive skill acquisition (21, 24, 30).

Motor and cognitive functions are both controlled by cerebellum, basal ganglia, and frontal lobe regions of the brain that cooperatively network to exercise control over the intentionality of movement and on executive functions that require the individual to anticipate and predict the requisite movements. Dysfunction associated with developmental delays all involve conditions of reduced optimization of brain integration that in turn disrupt executive function, frontal lobe-based behaviors, and articulation with motor components of the nervous system (22, 28, 31).

Not uncommonly seen in children with developmental delays, is motor incoordination or “clumsiness,” relating to posture and gait that supports the concept of “weak central coherence” that in turn relates to a processing bias for local and featural information and difficulty in extracting the essence of meaning, detecting missing or hidden figures or “seeing the big picture” in daily life experiences (22, 28, 32).

Among the limitations of this study included the fact that missing was information about the ethnicity of the participants. Among the elementary school participants, the students were of mixed ethnic background, but in the high-school population, all were Arabic speaking. Cultural effects are important issues to examine in future research. Additionally, mathematical skills as measured by PISA questions were only examined among high-school students and not among elementary school participants. In addition, a broad range of academic and cognitive skills were not examined in detail such as reading comprehension, reaction time, sensory-motor abilities, and the like. These are also the subjects of future research.

The tasks in this study simply examined the gross relation between walking as a form of motricity and feature detection and attention, cognitive, and academic performance. The results demonstrated the relationship and benefit between movement, in the form of walking, a postural function, and cognitive skills, such as critical feature detection.

## Conclusion

The public health implications of the relationship between children’s lifestyle and cognitive–motor interaction are significant. There exists a pandemic of physical inactivity among all age groups. We have predictions that sedentariness will continue to grow throughout the world in the near and long-term future (33). We can conclude that at least 10 min of walking has significant effect on mathematical problem-solving abilities in higher performing high school, but more so in lower performing students according to the results of testing mathematical problem-solving ability according to the Bloom’s Taxonomy. Additionally, in all age groups 10 min of walking is a minimum that can affect performance in Simon-type memory and feature-detection tasks independent of children’s age.

## Ethics Statement

This study was carried out in accordance with the recommendations of the Research Committee of Oranim Academic College with written informed consent from all participant’s parents or guardians. All subjects’ parents or guardians gave written informed consent in accordance with the Declaration of Helsinki. The protocol was approved by the Research Committee of Oranim Academic College.

## Author Contributions

GL and RM wrote the paper. YZ, OM, SG, OM, SK-M, MA, and AK collected data and participated in the experimental design. RM, GL, and AO performed the statistical analyses.

## Conflict of Interest Statement

The authors declare that the research was conducted in the absence of any commercial or financial relationships that could be construed as a potential conflict of interest.
